# Incidence of postoperative pulmonary complications in patients undergoing minimally invasive versus median sternotomy valve surgery: propensity score matching

**DOI:** 10.1186/s13019-021-01669-7

**Published:** 2021-10-09

**Authors:** Mohamed Abdulkadir Mohamed, Cai Cheng, Xiang Wei

**Affiliations:** Division of Cardiothoracic and Vascular Surgery, Tongji Hospital, Tongji Medical College, Huazhong University of Science and Technology, 1095# Jiefang Ave., Wuhan, 4300 People’s Republic of China

**Keywords:** Minimally invasive surgery, Mitral valve, Aortic valve, Postoperative pulmonary complications, Full sternotomy

## Abstract

**Objective:**

Postoperative pulmonary complications (PPCs) are common incidents associated with an increased hospital stay, readmissions into the intensive care unit (ICU), increased costs, and mortality after cardiac surgery. Our study aims to analyze whether minimally invasive valve surgery (MIVS) can reduce the incidence of postoperative pulmonary complications compared to the full median sternotomy (FS) approach.

**Methods:**

We reviewed the records of 1076 patients who underwent isolated mitral or aortic valve surgery (80 MIVS and 996 FS) in our institution between January 2015 and December 2019. Propensity score-matching analysis was used to compare outcomes between the groups and to reduce selection bias.

**Results:**

Propensity score matching revealed no significant difference in hospital mortality between the groups. The incidence of PPCs was significantly less in the MIVS group than in the FS group (19% vs. 69%, respectively; *P* < 0.0001). The most common PPCs were atelectasis (*P* = 0.034), pleural effusions (*P* = 0.042), and pulmonary infection (*P* = 0.001). Prolonged mechanical ventilation time (> 24 h) (*P* = 0.016), blood transfusion amount (*P* = 0.006), length of hospital stay (*P* < 0.0001), and ICU stay (*P* < 0.0001) were significantly less in the MIVS group. Cardiopulmonary bypass (CBP), aortic cross-clamping, and operative time intervals were significantly longer in the MIVS group than in the matched FS group (*P* < 0.001). A multivariable analysis revealed a decreased risk of PPCs in patients undergoing MIVS (odds ratio, 0.25; 95% confidence interval, 0.006–0.180; *P* < 0.0001).

**Conclusion:**

MIVS for isolated valve surgery reduces the risk of PPCs compared with the FS approach.

## Introduction

The full sternotomy approach has been the gold standard for cardiac valve repair or replacement for many years, and most cardiac surgical centers continue to use it because it allows excellent exposure of the heart and great vessels. However, minimally invasive surgery has been undergoing rapid development in the last few decades. The technique of minimally invasive valve surgery (MIVS) was introduced to reduce surgical trauma and bleeding, decrease postoperative pain, and promote earlier discharge and quicker postoperative recovery [1–3].

These findings, along with a decrease in-hospital mortality in elderly patients, lead to an increase in the acceptance of minimally invasive valve surgery as an alternative to traditional full median sternotomy surgery in many centers worldwide [4]. Despite such improvements in surgical management and patients’ care over the years, however, pulmonary complications remain a leading cause of morbidity and mortality after cardiac surgery [5, 6]. These complications contribute to longer hospital stays and more readmissions into the ICU, significantly affecting health care and increasing the financial burden to health care systems [7, 8].

The most common pulmonary complications include atelectasis and pleural effusions, pneumonia, pneumothorax, diaphragm paralysis due to phrenic nerve injury, and pulmonary infection [6]. Whereas the association between full median sternotomy surgery and development of postoperative pulmonary complications has been widely established, however there is still ongoing debate about the effect of MIVS on postoperative pulmonary complications (PPCs), and the associations remain unclear. To the best of our knowledge, no previous clinical study has directly compared the incidence of pulmonary complications among matched groups after minimally invasive valve surgery vs. full median sternotomy. Therefore, our study aimed to analyze the effect of MIVS on the incidence of PPCs in patients undergoing isolated mitral or aortic valve surgery. We analyzed the outcomes of patients who underwent MIVS and compared them with a cohort that underwent surgery via an FS approach.

## Material and methods

### Patients selection and data collection

In our institution, 1076 patients underwent isolated mitral or aortic valve surgery through a minimally invasive technique or a full median sternotomy between January 2015 and December 2019. After we obtained institutional review board approval for the study and the requirement for patients' consent waived, the patients' data were retrospectively collected and analyzed.

All patients with isolated mitral valve or aortic valve disease who required valve surgery were eligible for this study. Patients who underwent concomitant coronary artery bypass grafting, ascending aortic surgery, atrial fibrillation ablation, or atrial septal defect repair and procedures other than isolated mitral and aortic valve surgery were excluded. We performed propensity score matching to reduce selection bias, more accurate results and to address imbalances between the groups. Two matched cohorts of 74 patients each were based on a propensity score analysis. Based on the following features, propensity scores were then calculated using a multivariate logistic regression model: age and sex; body mass index; smoking history; hypertension; diabetes mellitus; chronic obstructive pulmonary disease; existing cerebrovascular disease; left ventricular ejection fraction; atrial fibrillation; and New York Heart Association class III or IV.

### Definitions

Minimally invasive valve surgery (MIVS) was defined as any mitral or aortic valve surgery performed through a limited incision other than a full median sternotomy. In-hospital mortality was defined as any death that occurred during the hospitalization following surgery, regardless of cause. Postoperative stroke was defined as the appearance of a new neurologic symptomatic deficit, occurring more than 72 h after surgery. PPCs were defined as complications occurring in the postoperative period and producing clinical diseases such as atelectasis, pneumonia, pleural effusion, pneumothorax, pulmonary infection, and diaphragm dysfunction due to phrenic nerve injury [7]. Atelectasis, pneumonia, or pleural effusion were documented by a radiologist or physician based on chest radiography or a computed tomography scan. Prolonged ventilation time was defined as a ventilation period of more than 24 h. The definitions and variables selected were based on those in the Society of Thoracic Surgeons Database.

### Surgical techniques for MIVS

All patients were placed in the supine position, underwent anesthetic induction, and were intubated with a single-lumen endotracheal tube. Each patient had a Swan-Ganz catheter and a radial arterial line placed. The transesophageal echocardiography (TEE) probe was placed intraoperatively as a guide for evaluation of diseased valves and to assess postoperative outcomes regardless of the surgical procedure. External defibrillator pads were positioned behind the right scapula and in the apical area before sterilization. In all cases, the procedure began with exposing both the femoral artery and vein to establish cardiopulmonary bypass; a 3-cm incision was then made in the right groin area by placing a purse-string suture. After heparinization, the Seldinger wire technique was used to cannulate the femoral vessels under TEE guidance and a venous cannula was placed in the superior vena cava. If peripheral vascular disease was present, we used central ascending aortic cannulation for the replacement of the aortic valve, while for mitral valve procedures, we planned to use axillary artery cannulation, but it was not required in any of the patients.

For the video-assisted mitral valve procedures, the patients were positioned in a reverse Trendelenburg supine position with the right side of the chest slightly elevated 30° to 45° and rotated to the left. This position maximizes the clear visualization of the mitral valve through the incision. A 5- to 7-cm skin incision was made overlying the fourth to fifth intercostal space medial to the anterior axillary line to gain access to the thoracic cavity. Once thoracotomy was completed, additional stab incisions were used for the introduction of supportive instruments such as a thoracoscope and left ventricular vent, CO_2_ insufflator, and other pericardial stay sutures. After the pleural space was opened, a soft tissue retractor followed by a rib spreader was inserted to gently expand the intercostal space for further exposure. After pericardiotomy, the right atrium was opened, and the atrial septum was incised and exposed to visualize the mitral valve, using a specially designed atrial retractor. The mitral valve procedure was performed under thoracoscopic video assistance. The mitral valve was inspected, replacement or repair was achieved in the standard fashion, and a 4-0 Prolene polypropylene suture was used to close the left atrium.

The aortic valve procedure was performed under direct vision through an incision of 5–6 cm, located at the second to third intercostal space. This technique minimized surgical trauma and provided adequate surgical exposure of the aorta without rib resection. The left ventricular vent was placed through the right superior pulmonary vein cannulas. A standard transverse aortotomy obtained access to the aortic valve under direct vision, the valve was removed and replaced in the standard fashion, and the pericardium was left open.

In all cases, CO_2_ continuous insufflation was used in the operative field and the right lung ventilation was deflated. The pericardium was opened 2–3 cm above the phrenic nerve and over the aorta to facilitate the exposure, and pericardial retractor sutures were applied and brought out of the thoracic cavity. Cardiopulmonary bypass (CPB) was established, and the left ventricular vent was placed into the left ventricle via a purse-string suture through the right superior pulmonary vein cannulas. Aortic cross-clamping was achieved by using a flexible and retractable shaft cross-clamp before incising the right atrium. The cold antegrade blood cardioplegia solution was delivered directly to the aortic root or selectively through the coronary ostial cannulation via the aortic cannula. Before the patients were weaned from CPB, hemostasis was carefully ensured, and mediastinal drainage tubes were inserted. At the end of all procedures, the chest incision was closed in routine fashion, and the patients were transferred to the postoperative ICU and managed according to the institutional protocol. TEE was used routinely before and after weaning from the CPB machine to check proper cardiac valvular function, confirm de-airing procedure, and evaluate surgical results.

### Statistical analysis

Values for continuous variables were described as the mean ± standard deviation. The difference between the two groups was compared using Student’s *t*-test. Categorical variables were expressed as percentages or frequency distributions, and groups differences were assessed using the χ^2^ test or Fisher exact test, as appropriate. Propensity score matching was used to decrease the influence of selection bias between the groups. To evaluate the variables that were predictors of composite complications, multivariable logistic regression analysis was performed. A *P* value of < 0.05 was considered statistically different, and all statistical analyses were performed using SPSS version 26.0.

## Results

A total of 1076 patients were identified. Of these patients, 80 had valve surgery via a minimally invasive approach and 996 underwent a full sternotomy. The procedures were successful in both groups, and no surgical conversion from minimally invasive to full sternotomy occurred during surgery. No significant differences were found in baseline characteristics of the entire cohort (Table 1).

**Table 1:** Preoperative characteristics of study groups before and after matching

	Overall unmatched patients			Overall patients after propensity score-matching			Patients after propensity score-matching					
							Mitral valve			Aortic valve		
Variables	MIVS	FS	*p* value	MIVS	FS	*p* value	Minimally invasive	FS	*p* value	Minimally invasive	FS	*p* value
	n = 80	n = 996		n = 74	n = 74		n = 42	n = 42		n = 29	n = 29	
Age (years)	51.1 ± 15.9	50.9 ± 11.98	0.959	51.35 ± 16.2	50.8 ± 13.12	0.824	46.0 ± 12.1	48.34 ± 10.42	0.341	60.48 ± 15.5	55.6 ± 8.8	0.142
Gender (Male)	47 (58.8)	550 (55.5)	0.541	44 (59.5)	43 (58.1)	0.867	24 (57.1)	15 (35.7)	0.49	19 (65.5)	16 (55.2)	0.421
BMI, (kg/m2)	23.47 ± 4.72	22.19 ± 4.02	0.007	23.6 ± 4.84	22.21 ± 4.26	0.065	24.15 ± 5.6	21.3 ± 3.5	0.006	23.4 ± 3.2	22.3 ± 4.1	0.265
Current or former smoker	18 (22.5)	74 (7.4)	0.0001	13 (17.6)	23 (31.1)	0.055	8 (19.0)	13 (31.0)	0.208	3 (10.3)	9 (31.0)	0.052
Hypertension	23 (28.7)	255 (25.6)	0.536	22 (29.7)	22 (29.7)	1.000	14 (33.3)	10 (23.8)	0.334	7 (24.1)	10 (34.5)	0.387
Diabetes Mellitus	9 (11.3)	65 (6.5)	0.108	9 (12.2)	10 (13.5)	0.806	3 (7.1)	2 (4.8)	0.645	6 (20.7)	6 (20.7)	1.000
COPD	9 (13.3)	61 (6.1)	0.074	4 (5.4)	11 (14.9)	0.057	3 (7.1)	7 (16.7)	0.178	5 (17.2)	9 (31.0)	0.220
History of Cerebrovascular disease	1 (1.3)	19 (1.9)	0.675	1 (1.4)	7 (9.5)	0.029	1 (2.4)	0 (0.0)	0.314	0 (0.0)	1 (3.4)	0.313
LVEF	64.38 ± 7.8	62.93 ± 6.9	0.072	63.9 ± 7.9	62.30 ± 7.3	0.178	65.07 ± 7.4	64.7 ± 7.5	0.815	61.8 ± 8.5	61.0 ± 6.8	0.686
Atrial fibrillation	4 (5.0)	11 (1.1)	0.004	18 (24.3)	23 (31.1)	0.358	3 (7.1)	8 (19.0)	0.106	1 (3.4)	1 (3.4)	1.000
NYHA Class												
I-II	58 (72.5)	634 (63.7)	0.112	54 (73)	40 (54.1)	0.017	33 (78)	27 (64.3)	0.147	20 (69)	22 (75.9)	0.557
III-IV	22 (27.5)	362 (36.3)		20 (27.0)	34 (45.9)		9 (21.4)	15 (35.7)		9 (31.0)	7 (24.1)	

Continuous variables are described by mean ± SD and by median, and categorical variables are shown as n (%)

*BMI* body mass index, *LVEF* left ventricular ejection fraction, *COPD* chronic obstructive pulmonary disease, *NYHA* New York Heart Association functional classification

Using propensity score matching in the entire cohort of patients, we identified 74 pairs of patients for 1:1 matching between MIVS and FS groups. As presented in Table 1, there were no significant differences between the two matched groups regarding the preoperative baseline characteristics.

### Intraoperative data

Patients who underwent MIVS had a significantly longer CBP, aortic cross-clamping, and operative times (120.55 ± 32.3 min vs. 101.89 ± 35.5 min, *P* < 0.001; 90.1 ± 20.4 min vs. 76.5 ± 19.1 min, *P* < 0.0001, 306.42 ± 101.1 vs. 252.9 ± 89.3 min, *P* < 0.001, respectively) (Table 2).

**Table 2:** Intraoperative and postoperative characteristic according to the study groups after matching

	Overall patients after propensity score-matching			Patients after propensity score-matching					
				Mitral valve			Aortic valve		
Variables	MIVS	FS	*p* value	Minimally invasive	FS	*p* value	Minimally invasive	FS	*p* value
	n = 74	n = 74		n = 42	n = 42		n = 29	n = 29	
Aortic Cross-clamp time, min	90.1 ± 20.4	76.5 ± 19.1	0.0001	85.2 ± 24.79	60.9 ± 20.97	0.0001	93.3 ± 19	77.03 ± 31	0.019
CBP time, min	120.55 ± 32	101.89 ± 35.5	0.001	119.6 ± 30.5	95.1 ± 20.4	0.0001	123.5 ± 55	109.8 ± 34	0.217
Operative time, min	306.42 ± 11	252.9 ± 89.3	0.001	295.3 ± 102.8	230.2 ± 118.9	0.0001	304.8 ± 94	249.1 ± 112	0.042
ICU length of stay, days	2.5 ± 1.9	4.8 ± 2.6	0.0001	2.83 ± 2.94	3.8 ± 2.97	0.143	2.79 ± 2.0	4.07 ± 2.2	0.029
Hospital stays, days	8.65 ± 2.6	12.6 ± 5.01	0.0001	8.5 ± 11.9	11.98 ± 3.51	0.0001	9.57 ± 2.4	16.17 ± 5.5	0.002
Transfusion RBCs of patients	13 (17.6)	28 (37.8)	0.006	11 (26.2)	21 (50.0)	0.025	5 (41.7)	7 (58.3)	0.517
Prolonged mechanical ventilation time (> 24 h)	7 (9.5)	18 (24.3)	0.016	3 (7.1)	12 (28.6)	0.010	4 (23.5)	13 (76.5)	0.009

Continuous variables are described by mean ± SD and by median, and categorical variables are shown as n (%)

*CPB* cardiopulmonary bypass time, *ICU* intensive care unit, *RBC* red blood cells

### Postoperative outcomes

In-hospital clinical outcomes after propensity score matching are presented in Tables 2 and 3. The incidence of PPCs was significantly lower in the MIVS group than in the FS group (18.9 vs. 68.9, *P* = 0.0001, respectively). These complications included pulmonary infection (4.1% vs. 21.6%, *P* = 0.001), pleural effusion (4.1% vs. 13.5%, *P* = 0.042), and atelectasis (5.4% vs. 16.2%, *P* = 0.034), respectively. Although patients in the MIVS group had a lower incidence of pneumonia, pneumothorax, and phrenic nerve injury than those in the FS group, no significant differences were found for these other PPCs between the groups. Table 3 presents the significance of the total incidence of PPCs.

**Table 3:** Postoperative characteristics of patients and pulmonary complications according to the study groups after matching

	Overall patients after propensity score-matching			Patients after propensity score-matching					
				Mitral valve			Aortic valve		
Variables	MIVS	FS	*p* value	Minimally invasive	FS	*p* value	Minimally invasive	FS	*p* value
	n = 74	n = 74		n = 42	n = 42		n = 29	n = 29	
In Hospital mortality	1 (1.4)	2 (2.7)	0.513	0 (0.0)	4 (9.5)	0.040	2 (6.9)	3 (10.3)	0.640
Stroke	1 (1.4)	4 (5.4)	0.172	0.0	0.0	–	1 (3.4)	2 (6.9)	0.553
Pulmonary complications	14 (19)	51 (69)	0.0001	13 (31.0)	38 (90.5)	0.0001	12 (41.4)	23 (79.3)	0.003
pneumonia	2 (2.7)	4 (5.4)	0.405	1 (2.4)	0 (0.0)	0.314	0 (0.0)	0 (0.0)	–
pneumothorax	1 (1.4)	4 (5.4)	0.172	0.0	0.0	–	1 (3.4)	4 (13.8)	0.160
Atelectasis	4 (5.4)	12 (16.2)	0.034	5 (11.9)	13 (31.0)	0.033	2 (6.9)	12 (41.4)	0.002
phrenic nerve injury	1 (1.4)	3 (4.1)	0.311	1 (2.4)	0 (0.0)	0.314	0 (0.0)	1 (3.4)	0.313
pleural effusion	3 (4.1)	10 (13.5)	0.042	2 (4.8)	4 (9.5)	0.397	1 (50.0)	1 (50.0)	1.000
Pulmonary infection	3 (4.1)	16 (21.6)	0.001	1 (2.4)	6 (14.3)	0.048	2 (6.9)	1 (3.4)	0.553
Tracheostomy	0 (0.0)	2 (2.7)	0.154	0 (0.0)	3 (7.1)	0.078	1 (3.4)	0 (0.0)	0.313

Continuous variables are described by mean ± SD and by media, and categorical variables are shown as n = number of patients and %

Compared with patients in the FS group, those in the MIVS group had significantly shorter lengths of ICU and postoperative hospital stays (2.5 ± 1.9 days vs. 4.8 ± 2.6 days, *P* < 0.0001, 8.65 ± 2.6 days vs. 12.6 ± 5.01 days, *P* < 0.0001, respectively). Also, in comparison with the FS group, the MIVS group had a significantly lower incidence of patients required of red blood cell transfusion and shorter postoperative ventilation time (17.6% vs. 36.8%, *P* = 0.006, 9.5% vs. 24.3%, *P* = 0.016, respectively). Although patients in the MIVS group had a slightly lower incidence of mortality and postoperative stroke than those in the FS group, no significant difference was found in these parameters between the two groups (1.4% vs. 2.7%, *P* = 0.513; 1.4% vs. 5.4%, *P* = 0.172, respectively).

For the mitral valve patients, in-hospital mortality was 0 (0.0%) for the minimally invasive group and 4 (9.5%) for the median sternotomy group (*P* = 0.04). The groups showed no difference in stroke after matching. Patients undergoing minimally invasive mitral valve surgery had a shorter length of hospital stay and a lower incidence of blood transfusion than those in the FS group (8.5 ± 11.9 days vs. 11.98 ± 3.51 days, *P* = 0.0001; 26.2% vs. 50.0%, *P* < 0.025, respectively) (Table 2). Significant differences were found in operative time, aortic cross-clamping time, and CBP time (295.6 ± 102.8 min vs. 230.2 ± 118.9 min, *P* < 0.0001; 85.2 ± 24.79 min vs. 60.90 ± 20.97 min, *P* < 0.0001, 119.6 ± 30.5 min vs. 95.1 ± 20.4 min, *P* < 0.001, respectively). The FS group was associated with increased early PPCs (*P* = 0.0001) and longer mechanical ventilation time (*P* = 0.010), as shown in Tables 2 and 3.

For the aortic valve patients, preoperative characteristics did not differ between groups (Table 1). There were no significant differences in median cardiopulmonary bypass and aortic cross-clamping time between groups (Table 2). Patients undergoing minimally invasive aortic surgery required a shorter ICU stay and had a shorter length of hospital stay. Table 3 outlines PPCs. There was lower incidence of PPCs in the minimally invasive group (12/29, 41%) than in the FS group (23/29, 79%) (*P* = 0.003). In-hospital mortality and postoperative stroke rate were not significantly different between the groups.

In the entire cohort of matched patients, 65 developed PPCs and 83 did not. With regard to preoperative characteristics, only chronic obstructive pulmonary disease and cigarette smoking had a significant relationship with PPCs (18.5% vs. 3.6%, *P* < 0.003 and 33.8% vs. 16.9%, *P* < 0.017, respectively). There were no significant differences for age, sex, body mass index, hypertension, diabetes, and previous cerebrovascular disease with regard to PPC incidence (Table 4).

**Table 4:** Patients with postoperative pulmonary complications (PPCs) versus without PPCs

Variables	With PPCs	Without PPCs	*p* value
	n = 65	n = 83	
Age (years)	51.4 ± 12.4	50.9 ± 16.38	0.834
Gender (male)	35 (53.8)	52 (62.7)	0.280
BMI, (kg/m^2^)	22.75 ± 5.9	23.03 ± 3.2	0.725
LVEF	62.74 ± 7.2	63.46 ± 7.8	0.566
COPD	12 (18.5)	3 (3.6)	0.003
Hypertension	20 (30.8)	24 (28.9)	0.807
Diabetes	8 (12.3)	11 (13.3)	0.865
Atrial fibrillation	15 (23.1)	26 (31.3)	0.266
Current or former smoker	22 (33.8)	14 (16.9)	0.017
Aortic Cross-clamp time, min	85.9 ± 21.7	81.3 ± 20.1	0.187
CBP time, min	116.43 ± 31.8	107.1 ± 37.1	0.110
Operative time, min	301.7 ± 74.6	262.5 ± 111.7	0.016
ICU length of stay, days	4.4 ± 2.6	3.1 ± 2.4	0.001
Hospital stay, days	11.88 ± 5.3	9.6 ± 3.4	0.002
Transfusion RBC’s of patients	25 (38.5)	16 (19.3)	0.010
Prolonged mechanical ventilation time (> 24 h)	16 (24.6)	9 (10.8)	0.026
Hospital mortality	3 (4.6)	0 (0.0)	0.048

Continuous variables are described by mean ± SD and by median, and categorical variables are shown as n (%)

*BMI* body mass index, *LVEF* left ventricular ejection fraction, *COPD* chronic obstructive pulmonary disease, *NYHA* New York Heart Association functional classification, *CPB* cardiopulmonary bypass time, *ICU* intensive care unit, *RBC* red blood cells

For operation data, only operation time was associated with PPC. The time was significantly higher in patients with PPC than in patients without PPC (301.7 ± 74.6 min vs. 262.5 ± 111.7 min, *P* = 0.016, respectively). Neither aortic cross-clamping time nor CBP time was significantly different between the two groups.

Furthermore, patients with PPCs had a higher incidence of prolonged ventilation time (≥ 24 h) (24.6% vs. 10.8%, *P* = 0.026), blood transfusion (38.5% vs 19.3%, *P* = 0.10), longer ICU stay (4.4 ± 2.6 days vs. 3.1 ± 2.4 days, *P* = 0.001), and hospital stay (11.88 ± 5.3 days vs. 9.6 ± 3.4 days, *P* = 0.002) compared with patients without PPCs. All patients who died after surgery had a PPC, and there was a significant relationship between death and PPCs (Table 4).

According to the multivariate analysis, minimally invasive surgery was associated with a reduction in PPCs (odds ratio [OR] 0.25, 95% confidence interval [CI] 0.006–0.180, *P* < 0.0001), while the presence of chronic obstructive pulmonary disease (OR 5.27, 95% CI 1.075–25.8, *P* = 0.004), prolonged ventilation time for 24 h or more (OR 1.704, 95% CI 1.080–2.69, *P* = 0.022), longer ICU duration (OR 1.300, 95% CI 1.087–1.555, *P* = 0.004), and prolonged operative time (OR 1.007, 95% CI 1.002–1.012, *P* = 0.011) were associated with an increased incidence of PPCs (Table 5).

**Table 5:** Multivariate logistic regression analysis of risk factors of postoperative pulmonary complications after surgery

Variable	Odd ratio value	Lower CI	Upper CI	*P* value
Minimally invasive surgery	0.25	0.006	0.180	0.0001
COPD	5.27	1.075	25.8	0.004
Prolonged ventilation time	1.704	1.080	2.690	0.022
Operative time	1.007	1.002	1.012	0.011
Duration of ICU stay	1.300	1.086	1.555	0.004

Hypertension, smoking, cardiopulmonary bypass time, aortic cross clamp time, and hospital duration and blood transfusion were included in the multivariable analysis and were not significant

*COPD* chronic obstructive pulmonary disease, *ICU* intensive care unit, *CI* confidence interval

## Discussion

Over the last few decades, there has been a gradual evolution in the practice of minimally invasive surgery with excellent postoperative outcomes [1, 9]. However, despite improved surgical techniques and preoperative management care, PPCs remain the leading cause of morbidity and mortality after cardiac surgery [10]. Furthermore, the development of PPCs after cardiac surgery is associated with an increased length of hospital stay and longer ICU admission, significantly affecting health care costs in cardiac surgery patients [11, 12]. No previous report described the incidence of postoperative pulmonary complications after minimally invasive valve surgery compared with full sternotomy. As a result, we evaluated the effects of minimally invasive valve surgery on the incidence of PPCs relative to conventional FS.

Our study demonstrated that in patients undergoing isolated valve surgery, a minimally invasive approach reduced the incidence of PPCs and had improved outcomes, although minimally invasive procedures had significantly longer aortic cross-clamping and CBP times and longer operative time.

The development of PPCs was previously associated with an increased incidence of blood transfusion and significantly longer ICU and hospital stays [10, 13]. Therefore, MIVS has significantly reduced the incidence of PPCs, which may have resulted in shorter hospital and ICU stays and reduced the need for blood transfusion.

The reduction in PPCs found with the minimally invasive approach may have been due to decreased trauma leading to reduced inflammation and a range of associated complications. We included the most common PPCs in our study (i.e., atelectasis, pneumonia, pleural effusion, pulmonary infection, and phrenic nerve injury). In addition, our study focused on patients with isolated valvular surgery who underwent MIVS or FS. However, more data are needed to form definite conclusions on the exact mechanism of this reduction.

Postoperative atelectasis has been reported to occur in 24% of patients after cardiac surgery, primarily in patients with postoperative pulmonary dysfunction, occurring in most patients with postoperative pulmonary dysfunction after cardiac surgery [14]. In the current study, 5.4% of patients experienced postoperative atelectasis after MIVS. Bonacchi et al. [15] reported an incidence of postoperative atelectasis of 7.5%. The lower incidence of atelectasis in our study may have been due to the use of positive end-expiratory pressure (7–8 cm H_2_O) and continuous positive airway pressure (5 cm H_2_O) during postoperative mechanical ventilation.

The increased stabilization of the thorax reported with minimally invasive surgery can help reduce the amount of pain that a patient experiences, shorten the period of analgesic use, help the patient perform coughing exercises, and enable early extubation [15–17]. Early extubation was previously linked with decreased rates of pulmonary complications after cardiac surgery, possibly due to improved respiratory dynamics, clearance of pulmonary secretion, coughing, earlier mobilization, and improved cardiac performance [18]. This improved recovery was confirmed in our study, with the MIVS group having a significant decrease in the incidence of prolonged ventilation time, leading to decreases in the duration of both ICU and hospital stays compared with the FS group. Therefore, these patients could achieve a more rapid postoperative recovery of pulmonary function and wean off mechanical ventilation earlier than those receiving FS. Reduced mechanical ventilation time in the ICU, as seen in the MIVS group, may lead to a decreased risk of ventilator-associated pneumonia and less airway trauma and facilitate earlier mobilization and early recovery [18]. Postoperative pneumonia remains the greatest threat to survival in a surgical patient, which is usually addressed together with atelectasis because many of the pathological changes provoked by atelectasis may predispose a patient to pneumonia. The reported incidence of pneumonia after cardiac surgery is 2.0–2.7% [19]. In our study, postoperative pneumonia occurred in (2.7%) of patients after matching.

Blood transfusions have also been reported to be associated with impaired postoperative pulmonary function and increased risk of PPCs, longer hospital stay, and higher costs for patients undergoing cardiac surgery [20]. Therefore, it is possible that, by decreasing the incidence of blood transfusions with MIVS, PPCs may be reduced. The overall results on PPCs suggest that the risks are lower when the approach is minimally invasive.

### Study limitations

Our study has several limitations despite its use of propensity score matching. This study is a single-center, retrospective study of a heterogeneous group of patients with a small sample size. Another limitation of this study is that all the included minimally invasive valve surgeries were performed through the right anterior mini-thoracotomy approach; therefore, the results cannot be extrapolated to represent outcomes obtained from other minimally invasive approaches. Finally, the findings of this study lacked a longer follow-up time, and the comparison of the surgical procedures was limited to in-hospital outcomes. A final determination would need a larger sample size and a multi-center study to determine whether MIVS reduces PPCs.

## Conclusion

Minimally invasive valve surgery is a safe approach that reduces surgical trauma and reduces postoperative pulmonary complications, resulting in shorter ICU and hospital stays. We believe our findings might help the surgeon's decision-making patient selection, especially when dealing with patients with a high risk of pulmonary diseases (such as COPD) undergoing cardiac valve surgery and are at increased risk of developing the pulmonary disease.

## Data Availability

The data are available from the corresponding author on reasonable requests.

## Abbreviations

CPB: cardiopulmonary bypass
COPD: chronic obstructive pulmonary disease
FS: full sternotomy
ICU: intensive care unit
MIVS: minimally invasive valve surgery
PPCs: postoperative pulmonary complications
TEE: transesophageal; echocardiography
